# Bidirectional interconversion of stem and non-stem cancer cell populations: A reassessment of theoretical models for tumor heterogeneity

**DOI:** 10.1080/23723556.2015.1098791

**Published:** 2015-12-02

**Authors:** Sanne M. van Neerven, Mathijs Tieken, Louis Vermeulen, Maarten F. Bijlsma

**Affiliations:** Laboratory for Experimental Oncology and Radiobiology, Center for Experimental and Molecular Medicine, Academic Medical Center, Amsterdam, The Netherlands

**Keywords:** Cancer stem cell, heterogeneity, interconversion, plasticity

## Abstract

Resolving the origin of intratumor heterogeneity has proven to be one of the central challenges in cancer research during recent years. Two theoretical models explaining the emergence of intratumor heterogeneity have come to dominate cancer biology literature: the clonal evolution model and the hierarchical/cancer stem cell model. Recently, a plastic model that combines elements of both the clonal and the hierarchical model has gained traction. Basically, this model proposes that cancer stem cells engage in bidirectional interconversion with non-stem cells, thereby providing the missing link between the 2 conventional models. Confirming bidirectional interconversion as a hallmark of cancer is a crucial step in understanding tumor heterogeneity and has important therapeutic implications. In this review, current methodologies and theoretical and empirical evidence regarding bidirectional interconversion will be discussed.

## Abbreviations

CSC: cancer stem cell
iPSC: induced pluripotent stem cell
NSCC: non-stem cancer cell
TME: tumor microenvironment

## Introduction

Our understanding of the molecular mechanisms underlying the biology of cancer has steadily increased over the last decades and, with it, the development of rationally designed targeted anticancer therapeutics. Unfortunately, the identification of potential therapeutic targets is hampered by the extensive heterogeneity observed *between* tumors (intertumor heterogeneity) and *within* tumors (intratumor heterogeneity). Currently, 2 models are used to explain intratumor heterogeneity: the clonal evolution model and the cancer stem cell (CSC) model.

The clonal evolution model, also referred to as the stochastic model, was first proposed in 1976 by Peter Nowell. He postulated that tumor development is a Darwinian process driven by the accumulation of spontaneous (epi-) genetic mutations followed by successive selection of clones.^1,2^ According to this theory, every cell is equally capable of becoming a cancer cell as long as it has acquired a competitive advantage over its neighbors. Ultimately, the cell population best suited for survival and proliferation is expected to dominate a tumor. Heterogeneity within this model is often attributed to microenvironmental influences and the presence of genetically distinct subclone populations.^3^ However, histologic analyses have revealed that tumors are often organized in a hierarchical fashion, a property that cannot be explained by the stochastic model. Therefore, a theory has emerged suggesting that only a subset of cells within a tumor is capable of tumor initiation and maintenance, and that these cells employ characteristics of healthy stem cells. Accordingly, these cells have been dubbed “cancer stem cells” (CSCs). Such cells have been identified in both leukemic and solid cancers.

The cancer stem cell model proposes that, much like normal tissue, tumors are organized in a hierarchical fashion, with rare multipotent and immortal CSCs at the top of the hierarchy, and transient, terminally differentiated non-stem cancer cells (NSCCs) forming the bulk of the tumor.^4–6^ According to this model, much of the observed tumor heterogeneity is the result of stable (epi)genetic regulation that is subjected to extensive intrinsic and extrinsic regulation.^7,8^ Currently, the CSC theory is seen as the prevailing model for tumor development as it most accurately explains the heterogeneity observed within tumors. In addition to contributing to heterogeneity, CSCs are suggested to be responsible for tumor progression, resistance to conventional chemotherapy, and increased invasiveness, and are therefore an interesting target for therapy. However, it is important to realize that the clonal and CSC models are not mutually exclusive in tumorigenesis: CSCs are able to undergo clonal expansion and selection and there is a clear role for the tumor microenvironment.^9–11^ Additionally, it is possible that only part of the tumor is organized hierarchically, while other sections are patterned by clonal evolution.^3^ Overall, however, the current models do not fully explain the observed heterogeneity, and the level of complexity might be greater than suspected.

Recently, a new type of model describing tumor heterogeneity has gained traction: the plastic CSC model. The plastic model combines elements of both stochastic and CSC theories. In agreement with the CSC model, it proposes that tumor heterogeneity is the result of hierarchical organization of phenotypic cell states. However, in contrast to the CSC model, it proposes that tumor populations behave in a dynamic fashion in which CSCs can differentiate into more mature progeny, and that differentiated cells may “dedifferentiate” back into stem-like cells, a process collectively referred to as *bidirectional interconversion*. Bidirectional interconversion is consequently linked to the clonal evolution model that proposes that non-tumorigenic cell fractions can in principle reacquire tumorigenic potential.

Bidirectional interconversion and dedifferentiation have been studied in many organisms and organ systems.^12,13^ In *Drosophila melanogaster*, differentiating germ cells can readily and stably revert into functional stem cells within the ovary.^14^ In mammals, this phenomenon has been described in organ systems such as the intestines,^15^ lungs,^16^ breast,^17,18^ and the heart.^19^ Dedifferentiation of committed progeny mostly occurs after tissue damage and implies that differentiated cells can function as a reserve to compensate for the loss of stem cells.^20^ This concept not only provides the basis for regenerative medicine, but also has major implications for cancer research. In particular, it affects our definition of what makes a stem cell. If bidirectional conversion contributes to tumorigenesis, it is essential to reassess our current models of tumor heterogeneity. In this review, we will discuss the principles of tumor plasticity and evaluate the involvement of bidirectional interconversion in tumor development.

## Considerations regarding the plastic model

**Figure 1.**
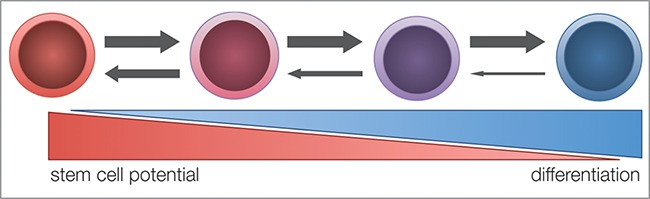
Stem cell potential hypothesis. Every cell is considered to possess a degree of stemness that is inversely correlated to its differentiation stage. This hypothesis predicts that non-stem cancer cells (NSCCs) with a high stemness potential are more likely to dedifferentiate into cancer stem cells (CSCs) than their more differentiated counterparts.

Critics of the plastic model argue that by introducing plasticity into conventional CSC models, the debate is at risk of becoming a purely semantic one. After all, if NSCCs can readily convert to CSCs, what is the use of drawing a functional distinction between the two?^21,22^ It is important to address some of the seemingly paradoxical properties of plastic models. Consider this: If a NSCC can in principle become a CSC, can a NSCC still be considered a mortal, non-tumorigenic cell? Would the boundary between NSCC and CSC become an arbitrary one? Instead, would it not be more practical to regard stemness as a cell property, where CSCs reside at the apex of the hierarchy and all other NSCCs lay on a spectrum of stem cell potential (Fig. 1)? One of the main arguments supporting this assumption is the inability to demonstrate phenotypically distinct CSC populations: purified CSC populations have always remained phenotypically heterogeneous, regardless of the selection criteria used.^23,24^ In principle, it can be envisioned that it is possible to use an ever more stringent and expansive set of criteria to allow for more phenotypically homogenous CSC populations;^25^ however, such criteria would most likely differ for each individual stem cell compartment. When viewed from the perspective of the plastic model, it makes less sense to search for a phenotypically distinct CSC population, as each individual cell is phenotypically fluid and will meet different criteria at different times. Instead, it might be more beneficial to define the parameters that govern the level of stemness in individual cells and assess how these can be influenced.

Although regarding stemness as a varying set of phenotypes is a compelling view, it should be noted that it is conceptually possible to accurately distinguish CSCs from NSCCs; however, it is wholly dependent on how we choose to functionally characterize CSCs. One could argue that the absolute minimal properties of a CSC are longevity and the capacity to divide asymmetrically.^26,27^ These features might apply to the whole stem cell compartment, but are more context dependent in the case of individual stem cells.^28,29^

## The dynamic relationship between CSC and NSCC populations

Within a tumor, cancer cells exist in various phenotypic states such as CSCs and NSCCs, and these states can influence the functional properties of the cell.^30^ It has become clear that these cell states are not static, but rather dynamic entities that are constantly remodeling. Although dynamic, the CSC/NSCC cell states are tightly regulated and restricted to a phenotypic equilibrium to ensure stable proportions of CSCs and NSCCs within a tumor.^31^ Evidently, when the proportion of CSCs/NSCCs is out of balance, the equilibrium can be restored by adapting the relative proliferative rates of the subpopulations.^31,32^ Stem cells have 2 possible modes of division: symmetric division, in which 2 daughter cells identical to the parent cell are produced, and asymmetric division, which creates a daughter with a stem cell fate and a cell destined for terminal differentiation.^33^ During asymmetric division the number of stem cells remains equal, whereas symmetric division results in an increase of the stem cell population relative to the total CSC/NSCC population.^34,35^ Symmetric stem cell division has been observed during development, and can persist into adulthood in normal tissue.^33^ Interestingly, bias toward symmetric division as a result of disrupted regulation of asymmetric division is associated with the formation of tumors.^36–38^

An alternative mechanism governing the CSC/NSCC equilibrium is bidirectional interconversion, which controls cell states by (de)differentiation of one state into the other. Evidence in favor of bidirectional interconversion is based on the appearance of CSCs in marker-sorted NSCC populations. However, there are some factors that need to be considered when studying bidirectional interconversion, such as the imperfect correlation between CSC markers and actual CSCs.^24^ For instance, CD133 has long been considered a colorectal CSC marker, but also appears to be expressed on differentiated progeny.^39^ The poor prognosis linked to increased CD133 expression is, in fact, related to hyperactivation of the MAP kinase pathway.^40^ Additionally, populations enriched for CSCs using molecular markers typically remain phenotypically heterogeneous and could also contain progenitors.^23,24^ Conversely, NSCC fractions could therefore also include CSCs. Indeed, in most studies using marker profiles to identify CSC populations, presumed NSCC fractions retain some degree of clonogenic and/or tumorigenic potential.^25,41-44^ Evidently, this imperfect correlation can be diminished by using an ever-increasing number of CSC markers.^25,45^

Additionally, gene expression noise can explain the appearance of CSCs in the NCSS population. Genes can switch between active and inactive states through intrinsically random transcriptional “bursts”, resulting in extensive variability in protein levels in clonal cell populations.^46^ Isogenic populations have been demonstrated to exhibit a wide variability in protein levels as a result of cell intrinsic gene expression noise.^47^ Therefore, a snapshot of gene expression levels in a large cell population—for example in FACS analysis—is expected to show a wide distribution of cells displaying high and low protein levels, irrespective of any hierarchical organization of phenotypic states within the population.^48^ Theoretically, if the transition between active and inactive transcriptional states of a gene is highly inefficient, mRNA and protein levels can become highly variable, resulting in a bimodal distribution of intracellular protein concentration at the population level.^48,49^ Single-cell analysis has revealed that gene expression noise does not control individual genes, but functions transcriptome-wide and reflects metastable states of reversible molecular lineage bias.^50,51^ Interestingly, a recent study has found that bimodal distributions of proteins at the population level can arise not only through inefficient transition between active and inactive transcriptional states, but also due to the collective spatial behavior of cell populations.^52^ Thus, in principle, transient gene expression stochastics and cell population effects can generate substantial fluctuations in the CSC/NSCC ratio that can mistakenly be interpreted as dedifferentiation. Therefore, when assessing bidirectional interconversion, these factors should be taken into account.

Importantly, it should be noted that proliferative expansion and bidirectional interconversion are not mutually exclusive mechanisms for CSC/NSCC dynamics; both may contribute to fluctuations in CSC population levels.^53^ Provided the plastic model is correct, it will be necessary to determine the relative contributions of both mechanisms to tumor development and recurrence. Additionally, it is essential to consider the drawbacks of marker-based experimental methods when studying phenotypic plasticity.

## Phenotypic plasticity, the factors that act on it, and its regulators

The concept of cellular plasticity has become reality ever since Yamanaka and colleagues generated induced pluripotent stem cells (iPSCs) from terminally differentiated somatic cells.^54^ Activation of just 4 transcription factors—Sox2, Oct-3/4, c-Myc, and KLF4—in murine fibroblasts could transform them into cells resembling embryonic stem cells (ESCs). Since the publication of this landmark paper, many variations on the “Yamanaka factors”, such as related transcription factors, miRNAs and small molecules, have been reported to dedifferentiate committed progeny into stem-like cells. It has become clear that the processes of regeneration and dedifferentiation are based on reactivation of developmental programs that are subjected to both genetic and epigenetic regulation. Importantly, it has been reported that reactivating developmental genes such as *c-Myc* and *KLF4* can lead to oncogenic transformation.^55^ This implies that similar mechanisms might be involved in tumorigenesis as in transformation of iPSCs.^56^ Indeed, genomic analysis of tumor samples often reveals mutations in genes encoding the Yamanaka factors or their downstream pathways, thereby altering intrinsic transcription regulation.^57–60^

For years, phenotypic plasticity during tumor development was by default attributed to the accumulation of (epi)genetic aberrations. This Darwinian model of mutation and selection follows a strict one-to-one genotype to phenotype pattern, where a certain genetic mutation results in a distinct phenotype. A mutation-induced dedifferentiated clone can subsequently be selected for. However, by doing so the Darwinian evolution model ignores the enormous variability of cell phenotypes that can be generated from a single genome and it is exactly this flexibility that enables reversible interconversion between differentiated and stem-like states in the absence of mutations.^61,62^ For instance, cancer cells in breast, lung, prostate, ovarian, and melanoma cancers are demonstrated to alter their gene expression profiles and transform into cell types that are not part of their original lineage.^61^ In addition, recent evidence suggests that mutations and subclones can accumulate in a neutral fashion without selective Darwinian sweeps.^63^ Mutations are then carried along and follow a more Lamarckian response, in which a better adapted phenotypic state is instructed by external stimuli.^64^ This concept is supported by observations of multiple subclones co-existing and reappearing within a tumor that according to Darwinian laws would have been outcompeted.^65^ Here, we will discuss external stimuli that influence cellular reprogramming and pressure cells to engage in bidirectional interconversion.

### The tumor stroma

Tumor development and progression is often closely associated with, and dependent on, its tumor microenvironment (TME). The TME comprises various cell types that support tumorigenesis by promoting tumor growth, angiogenesis, inflammation, and metastasis.^66^ In response to signals from the TME or tumor cells, healthy fibroblasts can transform into cancer-associated fibroblasts (CAFs).^67^ CAFs express growth factors including insulin like growth factor-II (IGF-II), hepatocyte growth factor (HGF), and vascular endothelial growth factor (VEGF), through which they increase tumor proliferation and support stemness in a Wnt- and Notch-dependent matter.^68^ In contrast to normal homeostasis in which Wnt and Notch signaling is strictly regulated and confined to the stem cell niche, TME-directed signaling can affect both CSCs and NSCCs. Since these signaling pathways regulate stemness, they can facilitate dedifferentiation of NSCCs into CSCs.^69^ For instance, HGF secreted by the TME can induce a more immature phenotype in differentiated intestinal tumor cells, including the expression of stem cell markers.^32^ Furthermore, HGF and other cytokines such as osteopontin (OPN) and stromal-derived factor 1α (SDF-1) are demonstrated to induce stem cell-associated glycoprotein CD44v6 expression in differentiated progeny.^70^ CD44v6 is associated with increased invasiveness and cell migration as a co-receptor of the HGF receptor MET, properties often attributed to CSCs.^71^ In addition, expression of TGF-β has been linked to dedifferentiation and epithelial-to-mesenchymal transition (EMT).^42^ Furthermore, mesenchymal cells within the TME can also promote stemness by expression of inflammatory chemokines and cytokines that result in activation of the NF-κB pathway.^72^

### Inflammation

Chronic inflammation is implicated in all steps of tumor development, from initiation and progression all the way to metastasis.^73^ In addition to the already heterogeneous infiltration of stromal cells, inflammation leads to an additional influx of immune cells. Pancreatic cancer is often preceded by persistent inflammation that is correlated with high invasiveness and early metastases to the liver and lung.^74^ Buchholz and colleagues have recently observed ectopic expression of NFATc1, originally identified as a T-cell transcription factor, in the majority of pancreatic cancers, especially those embedded in inflammatory niches.^75^ In addition, inflammation-induced NFATc1 forms chromatin complexes with STAT3 and accelerates transformation in *Kras*^G12D^ mutant cells.^76^ NFATc1 activation during embryogenesis is associated with EMT during lineage specification.^77^ Consistent with these findings, NFATc1 drives EMT through Sox2-mediated stemness (via upregulation of Snai1 and ZEB1) and is counteracted by levels of p53.^78^ Dedifferentiation has also been shown in murine prostate cancer models. Prostate epithelium is composed of basal and luminal lineages, which are self-sustained under normal conditions.^79^ Oncogenic transformation of luminal cells is preferred over basal cells, and it is proposed that basal cells can only generate tumors when dedifferentiated into luminal cells.^80^ In accordance, deletion of *PTEN* in K14-expressing basal cells results in prostate cancer with a long latency. Dedifferentiation from a basal to luminal phenotype is very rare and functions as a protective barrier against oncogenic transformation.^81^ However, bacteria-induced acute prostatitis results in tissue damage and promotes dedifferentiation of basal cells into luminal cells and thereby accelerates prostate neoplasia in the absence of *PTEN*.^82^ Inflammatory bowel disease (IBD), both hereditary and spontaneous, is a predictor for early development of colorectal cancer (CRC). NF-kB signaling is often enhanced in patients with IBD and correlates with a poor prognosis. Recently, Schwitalla and colleagues demonstrated that increased NF-kB signaling shortens survival and promotes dedifferentiation of epithelial intestinal cells by enhancing Wnt activation in this NSCC population.^83^ In melanoma cells, TNF-α secretion by macrophages promotes active interconversion between differentiated and non-differentiated cells in order to escape immune surveillance.^84^

### Therapy

There is a direct relationship between stemness and the reaction to stress stimuli. CSCs possess protective mechanisms and are therefore often implicated in therapy resistance.^85^ Indeed, an enrichment of the CSC population can be observed after therapy that frequently leads to tumor recurrence.^84,86^ Recent evidence suggests that, in addition to repopulation of the CSC pool by symmetric division, NSCCs can dedifferentiate and thereby also acquire resistance. One example is temozolomide (TMZ) treatment of glioblastoma. Despite the aggressiveness of this drug, relapse is often reported, presumably due to resistant CD133+ CSCs. Exposure of CSCs to TMZ leads to expansion of the stem cell pool, and exposure of differentiated cells to TMZ results in re-expression of CSC markers such as Sox2, Oct4, and Nestin *in vitro* and *in vivo*. This indicates that enrichment of the stem cell compartment is driven by both dedifferentiation and stem cell proliferation.^87^ In acute myeloid leukemia, vincristine treatment leads to epigenetic changes in the promoter region of the *MDR1* locus after chemotherapy.^88^ MDR1, or multidrug resistance protein 1, displaces a variety of drugs from the cell, thereby contributing to therapy resistance.^89^ Upregulation of MRD1 is considered to be a stem cell trait.^90,91^ Single-cell monitoring of a clonally generated cell population confirmed MDR1 expression in individual cells that correlated with Wnt signaling upregulation and could be reversed with β-catenin knockdown.^92^ In ovarian cancer cells, cisplatin, paclitaxel, or both agents combined induce stem-like characteristics and expression of CSC markers. These markers correlate with increased expression of ERCC1 and β-tubulin III, which are characteristic resistance proteins specific for platinum and taxane-based chemotherapeutics.^93,94^ Injection of these cells into the abdominal cavity of mice lead to a greater tumor burden.^95^ In addition, radiotherapy can also enhance expansion of the stem cell compartment by inducing a stem-like phenotype in non-stem breast cancer and prostate cells through Notch activation and upregulation of transcription factors such as Oct, Nanog, Klf4, and Sox2.^96,97^

### Hypoxia

Hypoxia has been implicated as a mediator of dedifferentiation in a number of solid tumors. In response to a lack of oxygen, an array of transcriptional responses is elicited via the hypoxia inducible factors (HIFs) HIF1-α and HIF-2α.^98^ In prostate cancer cells, hypoxic treatment resulted in stabilization of HIF1-α and HIF-2α, as well as upregulation of several transcription factors, including Nanog and Oct3/4.^99^ Pancreatic cancer cells show increased expression of Oct4 and c-Myc in response to hypoxia.^100^ In glioblastoma, CSCs appear to be more responsive to hypoxic conditions compared to NSCCs, with enhanced expression of a number of genes including HIF-2α and its transcriptional targets Oct4, Glut1, and Serpin.^101^ Hypoxic conditions have been demonstrated to facilitate the generation of iPSCs.^102^ Not only did hypoxic conditions yield a significant increase in the total number of reprogrammed cells, but reprogramming was achieved more quickly, increasing the percentage of transformed cells from 0.01% to ˜40% after 9 days. The rapid increase in reprogramming efficiency strongly suggests that the effects of hypoxia are not due to selective expansion of the transformed stem cells, but the result of an intracellular transcriptional response. Ma and colleagues reported increased colony formation capacity in prostate cancer cells cultured under hypoxic conditions for 48 h.^99^ Interestingly, proliferation rates were similar under normoxia and hypoxia, whereas G0/G1 phase was extended in hypoxic cells, indicating more cells in a quiescent state. Additionally, hypoxic treatment resulted in a 1.20- to 1.42-fold increase in ABCG2 transporters, and a 1.45- to 1.5-fold increase in CD44 expression within 48 h. CD44^high^ cells were confirmed to display greatly enhanced clonogenicity and sphere formation efficiency. Thus, the increase in CD44 expression and population stemness appears to be the result of cellular dedifferentiation, rather than population dynamics. Liang et al. found similar results in ovarian cancer cells, in which hypoxic conditions extended the G0/G1 phase and increased colony and sphere formation, accompanied by upregulation of CD44 and CD133.^103^ Interestingly, hypoxic treatment resulted in slower growth rates, whereas hypoxia pretreatment for 48 h yielded a significant increase in proliferation. Hypoxia clearly mediates stem cell function *in vitro*, most likely by cellular dedifferentiation rather than proliferative expansion of rare stem cells.

## Discussion

It is clear that the CSC/NSCC populations are not static within tumors. Guided by intrinsic and external signals, both populations are constantly remodeling but remain restricted to an equilibrium that is continuously restored. There are 2 mechanisms responsible for restoration of the equilibrium: (i) intercellular signals can modulate proliferation rates of distinct populations, or (ii) CSC/NSCC populations can engage in bidirectional interconversion.^31^ Although there is abundant evidence demonstrating differential proliferation rates between cell populations, the role of bidirectional interconversion is less well defined. Unfortunately, the study of stem cell dynamics is hindered by the inability to accurately separate the CSC and NSCC populations. This can be attributed to noisy gene expression levels or, more importantly, the lack of specific CSC markers. This implies that NSCC populations might contain CSCs that will either re-express their stem cell markers or express unidentified stem cell markers.

The paucity of accurate phenotypic markers, and therefore the inability to identify “true” CSCs, is in agreement with the plastic model that we have discussed. This model postulates that stemness is a cellular property, whereby CSCs simply reside at the peak of a hierarchical mountain of stem cell potential and NSCCs all possess a certain (but low) amount of stemness.^104,105^ Phenotypic markers could therefore overlap between CSCs and NSCCs with comparable stemness. The plastic model also provides an explanation for the variability in engraftment efficiency observed in tumor grafting studies,^25^ as NSCCs with a high(er) stem cell potential are assumed to be capable of grafting in experimental animals.^106^ Loh and Lim emphasize that the balance between stem cells and non-stem cells is delicate, and depends on competition of pluripotency factors and differentiation factors.^107^ Indeed, loss of single pluripotency factors often leads to differentiation to specific lineages, suggesting a connection between pluripotency and differentiation.^108^

**Figure 2.**
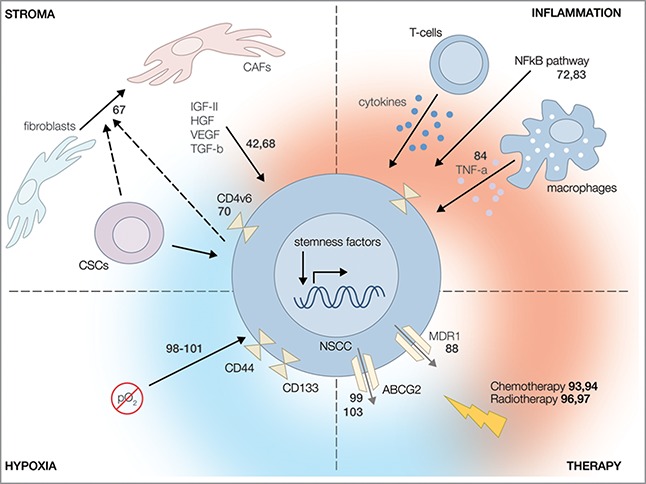
Regulators of phenotypic plasticity. There are several factors that promote bidirectional interconversion via activation of stemness factors. (Upper left panel) The TME and its accompanying cancer cells facilitate the transformation of healthy fibroblasts into cancer-associated fibroblasts (CAFs), which secrete growth factors that promote interconversion in a Wnt- and Notch-dependent matter. (Upper right panel) Inflammation and infiltrating immune cells can activate the NF-κB pathway in non-stem cancer cells (NSCCs) and thereby induce dedifferentiation into cancer stem cells (CSCs). (Lower left panel) Hypoxia promotes a more stem cell-like phenotype via enhanced activation of HIF-1α and HIF-2α factors. (Lower right panel) Cellular stress induced by therapy can influence expression of drug efflux pumps and results in therapy resistance in NSCCs characteristic of CSCs, thereby indicating dedifferentiation. Numbers indicate relevant references.

It seems reasonable that genetic aberrations can disturb this balance and induce dedifferentiation in NSCCs. In the case of the plastic model, NSCCs with a high stem cell potential are presumably more prone to dedifferentiation since they already express a more stem-like phenotype than their low-stemness neighbors. However, as tumor development is often associated with the accumulation of mutations^109^ it cannot be ruled out that more differentiated NSCCs can regain stem cell potential in a stepwise manner, as long as the right mutations are conferred. Besides mutation-induced interconversion, the TME can also play a role in phenotypic plasticity. The TME aims to provide the most optimal conditions for tumor development and regulates stemness by enhancing developmental pathways such as Wnt and Notch that can influence the differentiation state of cells (Fig. 2; upper left panel).^110^ Currently, CSC properties are often studied *in vitro*, removed from their native microenvironmental context. We should take into account that the absence of TME can potentially affect cellular behavior and tumorigenic potential.^111^ Moreover, the population equilibrium must be maintained *in vitro* to correctly study cell plasticity. It has been demonstrated that in the absence of stem cells differentiated cells are pressured to dedifferentiate, and direct contact with a single stem cell prevents this conversion.^112^ As a consequence, monocultures might not reflect normal CSC/NSCC population dynamics. Therefore, it will be important to study interconversion and cell plasticity within the context of their population equilibrium and the TME, for example in co-cultures and *in vivo* experiments.^113,114^

Furthermore, there is an explicit role for cellular stress in facilitating bidirectional interconversion that might provide a future therapeutic target.^85^ Bearing in mind the importance of healthy stem cells in tissue homeostasis, stem cells are usually well protected against intrinsic and extrinsic stress; they reside in a protective niche and exhibit upregulated stress responses and repair pathways compared to differentiated progeny. Moreover, they are more resistant to toxins as they display an increased drug transporter expression.^115^ In the case of stress within a tumor, such as hypoxia or infiltration of antitumor lymphocytes, stress stimuli can trigger dedifferentiation to provide NSCCs with a better stress response and thereby promote survival (Fig. 2; upper right panel and lower left panel). Dedifferentiation can also facilitate immunoescape, as interconversion alters the cell membrane markers by which NSCCs are recognized.^116^ Accordingly, bidirectional interconversion can also induce resistance to adaptive T-cell therapy, for example in melanoma.^84^ Similarly, phenotype plasticity can stimulate resistance to conventional chemotherapy and radiotherapy (Fig. 2; lower right panel). Therefore, it might be beneficial to reduce cellular stress and thereby decrease the chance of dedifferentiation, and thus therapy resistance. Indeed, reduction of inflammation by daily administration of nonsteroidal anti-inflammatory drugs (NSAIDs) can decrease tumor predisposition and incidence.^117^

To conclude, there is a clear involvement of bidirectional interconversion in tumorigenesis that can be influenced by both Darwinian and Lamarckian forces. At present, it is evident that all 3 proposed models of tumor heterogeneity contribute to tumor development. However, it remains unclear to what extent the individual models contribute to this process. Fortunately, recent advances in marker-free lineage techniques and deep sequencing methods enable us to determine these contributions and unravel clonal histories at a single-cell level. This will provide valuable information concerning the key mutations and environmental factors that influence cell plasticity. As cell plasticity and tumor stemness are directly associated with a poor prognosis, future challenges will be to develop more well-considered personalized therapeutic strategies aimed to predict and prevent bidirectional interconversion. Inhibition of cellular plasticity might sensitize cells for conventional treatments and subsequently reduce relapse.
